# The effect of VEGF, ET-1 and TGF-β1 levels in peritoneal dialysis effluent on peritoneal solute transport function

**DOI:** 10.3389/fmed.2025.1548218

**Published:** 2025-09-18

**Authors:** Xinxuan Han, Long Li, Xiangjiang Yu, Yan Jiang, Feifei Li, Shixi Luo, Xingxin Zeng, Ming Zhang

**Affiliations:** ^1^The 945th Hospital of the Joint Logistics Support Force of the Chinese People's Liberation Army, Ya'an, China; ^2^Ya'an Traditional Chinese Medicine Hospital, Ya'an, China; ^3^Yucheng District People's Hospital of Ya'an, Ya'an, China; ^4^Ya'an People's Hospital, Ya'an, China

**Keywords:** peritoneal dialysis effluent, vascular endothelial growth factor, Endothelin-1, transforming growth factor-β1, peritoneal solute transport function, prognosis

## Abstract

**Objective:**

This study aims to assess the impact of Vascular endothelial growth factor (VEGF), Endothelin-1 (ET-1), and Transforming growth factor-β1 (TGF-β1) levels in peritoneal dialysis effluent on peritoneal solute transport function.

**Methods:**

We included 450 patients from four hospitals, spanning from January 2016 to January 2018. Patients were categorized into low-transport (D/Pcr ≤ 0.65) and high-transport groups (D/Pcr > 0.65) based on solute transport rates. We compared the effluent levels of VEGF, ET-1, and TGF-β1 between the groups, employed Pearson’s correlation for analysis, and used multivariate logistic regression to identify independent factors influencing solute transport.

**Results:**

VEGF and TGF-β1 levels were significantly higher in the high-transport group (*p* < 0.05), showing a positive correlation with solute transport function (*r* = 0.721 and 0.539 respectively, *p* < 0.05). ET-1 levels showed no significant difference between the groups. VEGF and TGF-β1 were identified as significant independent factors affecting solute transport (OR = 3.438 and 3.684, *p* < 0.05). ROC curve analysis highlighted the predictive value of VEGF and TGF-β1 for solute transport function. Kaplan–Meier and COX regression analyses indicated that high transport was associated with lower survival rates, with VEGF and TGF-β1 serving as independent risk factors for mortality (RR = 3.442 and 3.550, *p* < 0.05).

**Conclusion:**

VEGF and TGF-β1 levels in peritoneal dialysis effluent are strongly correlated with solute transport function and serve as significant predictors of patient outcomes.

## 1 Introduction

The incidence of end-stage renal disease (ESRD) is increasing annually. Peritoneal dialysis (PD) is currently a primary renal replacement therapy, offering minimal hemodynamic impact while preserving residual renal function. This treatment modality utilizes the osmotic properties of a high-concentration dialysate and the semipermeable characteristics of the peritoneal membrane to remove retained metabolites, correct electrolyte and acid–base imbalances, and ultrafiltrate excess water (1, 2). Peritoneal membrane integrity remains crucial for sustained PD efficacy. However, long-term dialysate exposure induces mesothelial-mesenchymal transition (MMT), triggering pathological cascades including peritonitis and peritoneal fibrosis that culminate in ultrafiltration failure (3, 4). Chronic dialysate exposure induces MMT, serving as the pivotal trigger for pathological cascades that culminate in ultrafiltration failure. This process is driven by persistent biochemical stress from glucose degradation products (GDPs) and advanced glycation end-products (AGEs), which activate TGF-β/Smad, Wnt/β-catenin, and STAT3 signaling pathways in peritoneal mesothelial cells (5–8). The resultant MMT fuels dual pathology: First, it compromises peritoneal integrity through loss of tight junctions and glycocalyx degradation, exposing submesothelial tissue and triggering TLR4/NF-κB-mediated inflammation. Secretion of IL-6, TNF-α, and MCP-1 recruits neutrophils and macrophages, establishing recurrent peritonitis cycles that further damage the membrane. Second, transdifferentiated myofibroblasts overexpress α-SMA and orchestrate fibrogenesis via TGF-β-induced collagen deposition while recruiting resident fibroblasts. Concurrent VEGF-driven angiogenesis generates immature vasculature, elevating peritoneal solute transport rates. Collectively, these changes impair ultrafiltration through tripartite mechanisms: (1) Aquaporin-1 downregulation from glycocalyx damage cripples transcellular water transport; (2) Fibrotic thickening restricts small-solute diffusion and diminishes osmotic gradient efficacy; (3) Hyperperfusion from neovessels accelerates glucose absorption, collapsing the osmotic drive. This self-amplifying loop progressively compromises peritoneal function, ultimately manifesting as ultrafiltration failure.

Central to this process, transforming growth factor-β1 (TGF-β1) drives MMT in peritoneal mesothelial cells, converting them into myofibroblasts that secrete profibrotic mediators. This transition establishes the pathological basis for ultrafiltration failure through peritoneal neovascularization and fibrosis (9, 10). Mesothelial cells undergoing MMT produce key biomarkers: vascular endothelial growth factor (VEGF), the primary pro-angiogenic mediator increasing vascular permeability and fibrosis progression; and TGF-β1, a master pro-fibrotic regulator that amplifies its own production via autocrine signaling (5, 11). Additionally, endothelin-1 (ET-1) contributes to fibrotic injuries across tissues (12), with evidence suggesting mesothelial-derived ET-1 exacerbates peritoneal remodeling.

This study examines how VEGF, ET-1, and TGF-β1 levels in peritoneal effluent modulate solute transport function. Understanding MMT-mediated biomarker dynamics offers mechanistic insights for predicting and managing PD-related complications, ultimately improving patient outcomes.

## 2 Methods

### 2.1 General information

A total of 450 ESRD patients undergoing peritoneal dialysis from four public hospitals in Ya’an City were enrolled in this retrospective study from January 2016 to January 2018 (There were 64 cases in the 945 Hospital of the Joint Logistic Support Force of the Chinese People’s Liberation Army, 90 cases in Ya’an Traditional Chinese Medicine Hospital, 137 cases in the Yucheng District People’s Hospital of Ya’an, and 159 cases in the People’s Hospital of Ya’an). Comprising 255 males (56.7%) and 195 females (43.3%) with a mean age of 56.27 ± 9.34 years (range: 25–70 years). The primary regional distribution encompassed Ya’an City residents, though specific causes of ESRD and genetic variability data were not systematically documented in this retrospective cohort. Based on the Peritoneal Equilibration Test, patients were divided into two groups: the low-transport group (*n* = 205, D/Pcr ≤ 0.65) and the high-transport group (*n* = 245, D/Pcr > 0.65). The low-transport group comprised 110 males and 95 females, with a mean age of 56.37 ± 9.22 years (range, 25–70 years). The high-transport group included 145 males and 100 females, with a mean age of 56.14 ± 9.47 years (range, 25–70 years). No significant differences were found in the general demographic data between the two groups (*p* > 0.05), indicating that the groups were comparable.

This retrospective study was conducted with a waiver of patient informed consent. The Ethics Committee of the 945 Hospital of the Joint Logistics Support Force of the Chinese People’s Liberation Army approved the study (Ethics approval number: 202403018). All procedures were performed in accordance with the relevant requirements of the World Medical Association Declaration of Helsinki.

### 2.2 Inclusion and exclusion criteria

Inclusion criteria: (1) ESRD was confirmed by laboratory tests and renal color ultrasonography; (2) More than 3 months of regular peritoneal dialysis; (3) Peritonitis had not occurred in the previous 3 months; (4) Complete clinical data.

Exclusion criteria: (1) In the past 3 months, infection or adverse cardiovascular events; (2) Combined with malignant tumors; (3) The patients were suffering from a disease of the connective tissue.

### 2.3 Method of determination

Two groups of patients received 2.5% glucose dialysate overnight prior to the study. The following morning, the dialysate was drained, and peritoneal dialysate and venous blood samples were collected at 0, 2, and 4 h. Glucose and creatinine concentrations were measured in these samples. Creatinine concentration was corrected using a standard formula, and the ratio of the 4-h dialysate corrected creatinine concentration to the plasma corrected creatinine concentration (D/Pcr) was calculated to determine peritoneal transport function.

For the detection of VEGF, ET-1, and TGF-β1 levels, 10 mL of peritoneal dialysate was collected and processed by centrifugation using a H1650-W benchtop micro high-speed centrifuge (Jinan Boxin Biotechnology Co.) at a speed of 1,500 r/min for 5 min. The supernatant was retained and analyzed using enzyme-linked immunosorbent assay (ELISA) kits, which were purchased from Shanghai Enzyme-Linked Bio-Technology Co.

Patients were followed up every 6 months through telephone interviews and outpatient visits, with a primary focus on their survival status. The follow-up period concluded in December 2023.

### 2.4 Observation indicators

1. The levels of VEGF, ET-1, and TGF-β1 were compared in the peritoneal dialysis effluent of both low transport and high transport groups.
2. The correlation between levels of VEGF, ET-1, TGF-β1 in peritoneal dialysis effluent and peritoneal solute transport function was analyzed using the Pearson test.
3. Multivariate logistic regression was employed to examine the objective factors that have an impact on the peritoneal solute transport function.
4. The study utilized a Receiver Operating Characteristic (ROC) curve to evaluate the predictive capability of the levels of VEGF, ET-1, and TGF-β1 within peritoneal dialysis effluent in relation to peritoneal solute transport function.
5. The endpoint event was all-cause death. The survival rate of the low transport group and high transport group was evaluated using Kaplan–Meier survival analysis, while the COX proportional hazards model was utilized to analyze the risk factors that affect the patients’ prognosis.

### 2.5 Statistical methods

SPSS 24.0 software was employed to analyze and process the data. Statistical significance was inferred with *p* < 0.05. Measurement data were presented using ( $\bar{x}\pm s$ ) and compared with a *t*-test. The Pearson test was employed to examine the interrelationship between the concentrations of VEGF, ET-1, TGF-β1 in the peritoneal dialysis exudate and the function of solute transportation in the peritoneum. Multivariate logistic regression was utilized to explore the independent factors that influence peritoneal solute transport function. The predictive value of the levels of VEGF, ET-1 and TGF-β1 within peritoneal dialysis effluent concerning peritoneal solute transport function was analyzed through the utilization of a ROC curve. The survival rate of the low transport group and high transport group was evaluated using Kaplan–Meier survival analysis, and the risk factors affecting the patients’ prognosis were analyzed using the COX proportional hazards model.

## 3 Results

### 3.1 The levels of VEGF, ET-1 and TGF-β1 in peritoneal dialysis effluent of low transport group and high transport group

**Figure 1:**
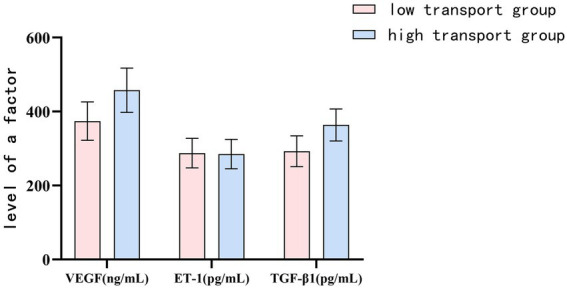
The levels of VEGF, ET-1 and TGF-β1 in peritoneal dialysis effluent of low-transport group and high-transport group.

The levels of VEGF and TGF-β1 in the peritoneal dialysis effluent were higher in the high transport group compared to the low transport group (*p* < 0.05), while there was no notable variance in ET-1 levels between the two groups (*p* > 0.05). Please refer to Table 1 and Figure 1.

**Table 1:** Comparison of levels of VEGF, ET-1 and TGF-β1 in peritoneal dialysate between low and high transport groups (Mean ± Standard Deviation).

Groups	VEGF (ng/mL)	ET-1 (pg/mL)	TGF-β1 (pg/mL)
Low transit group (*n* = 205)	374.11 ± 51.80	287.51 ± 39.95	292.73 ± 41.56
High transit group (*n* = 245)	457.56 ± 59.90	284.92 ± 39.77	363.74 ± 43.29
*t*-value	6.995	0.307	7.892
*p*-value	<0.001	0.760	<0.001

### 3.2 Analysis of correlation

**Figure 2:**
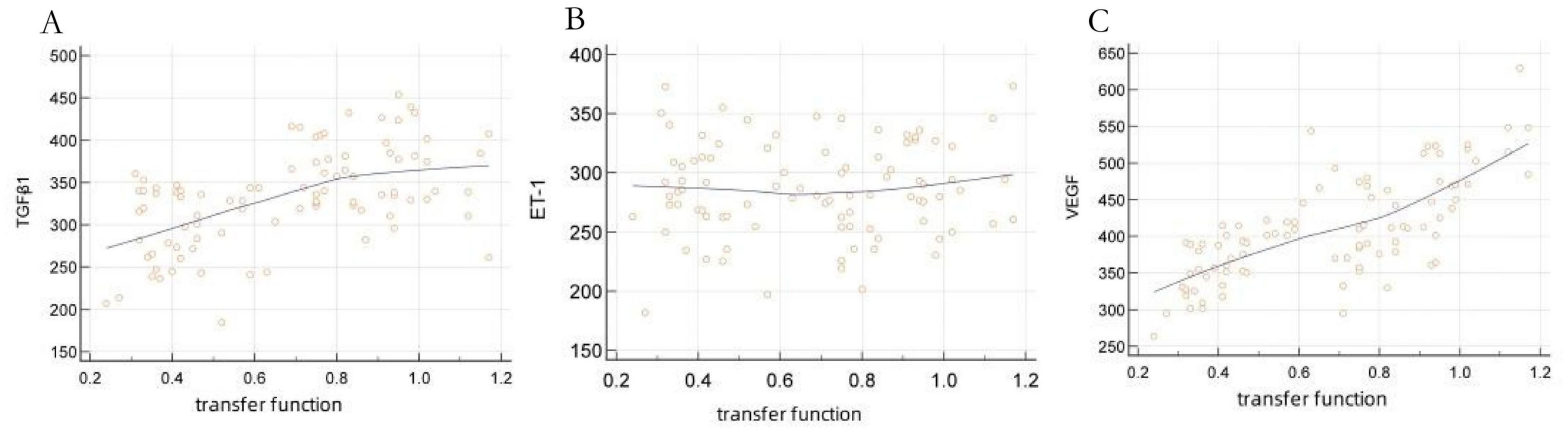
Scatter plots showing VEGF/TGF-β1 associations with solute transport rates. This visual representation specifically highlights the significant positive correlations of VEGF and TGF-β1, providing intuitive trend visualization that complements Table 2’s quantitative data.

The Pearson correlation test demonstrated a positive correlation between the levels of VEGF and TGF-β1 in the effluent obtained from peritoneal dialysis and the function of solute transport in the peritoneum (*r* = 0.721, 0.539, *p* < 0.05). Conversely, there was no noteworthy correlation between the level of ET-1 and peritoneal solute transport function (*p* > 0.05). Please refer to Table 2 and Figure 2.

**Table 2:** Pearson correlation coefficients of VEGF, ET-1 and TGF-β1 levels with peritoneal solute transport function in peritoneal dialysis effluent.

Indicators	Peritoneal transport function	
	*r-*value	*p*-value
VEGF	0.721	<0.001
ET-1	0.055	0.604
TGF-β1	0.539	<0.001

This table presents the complete statistical output of Pearson correlation analysis, including *r*-values and *p*-values for all three biomarkers. As shown, VEGF and TGF-β1 exhibited significant positive correlations (r = 0.721, 0.539; *p* < 0.001), while ET-1 showed no significance (*p* > 0.05). ral; A, Agree; SA, Strongly Agree.

### 3.3 Multivariate logistic regression analysis was conducted

Multivariate logistic regression analysis indicated that VEGF and TGF-β1 levels in the peritoneal dialysis effluent independently influenced the peritoneal solute transport function (OR = 3.438, 3.684, *p* < 0.05). Please refer to Table 3.

**Table 3:** Multivariate logistic regression analysis was employed to examine the determinants of peritoneal solute transport function.

Variant	*β* value	SE value	Wald *χ*^2^ value	*p*-value	OR value	95 percent CI
VEGF	1.235	0.417	8.771	0.003	3.438	1.518–7.786
ET-1	1.156	0.691	2.799	0.095	3.177	0.820–12.309
TGF-β1	1.304	0.493	6.996	0.008	3.684	1.402–9.682

### 3.4 ROC curve analysis

**Figure 3:**
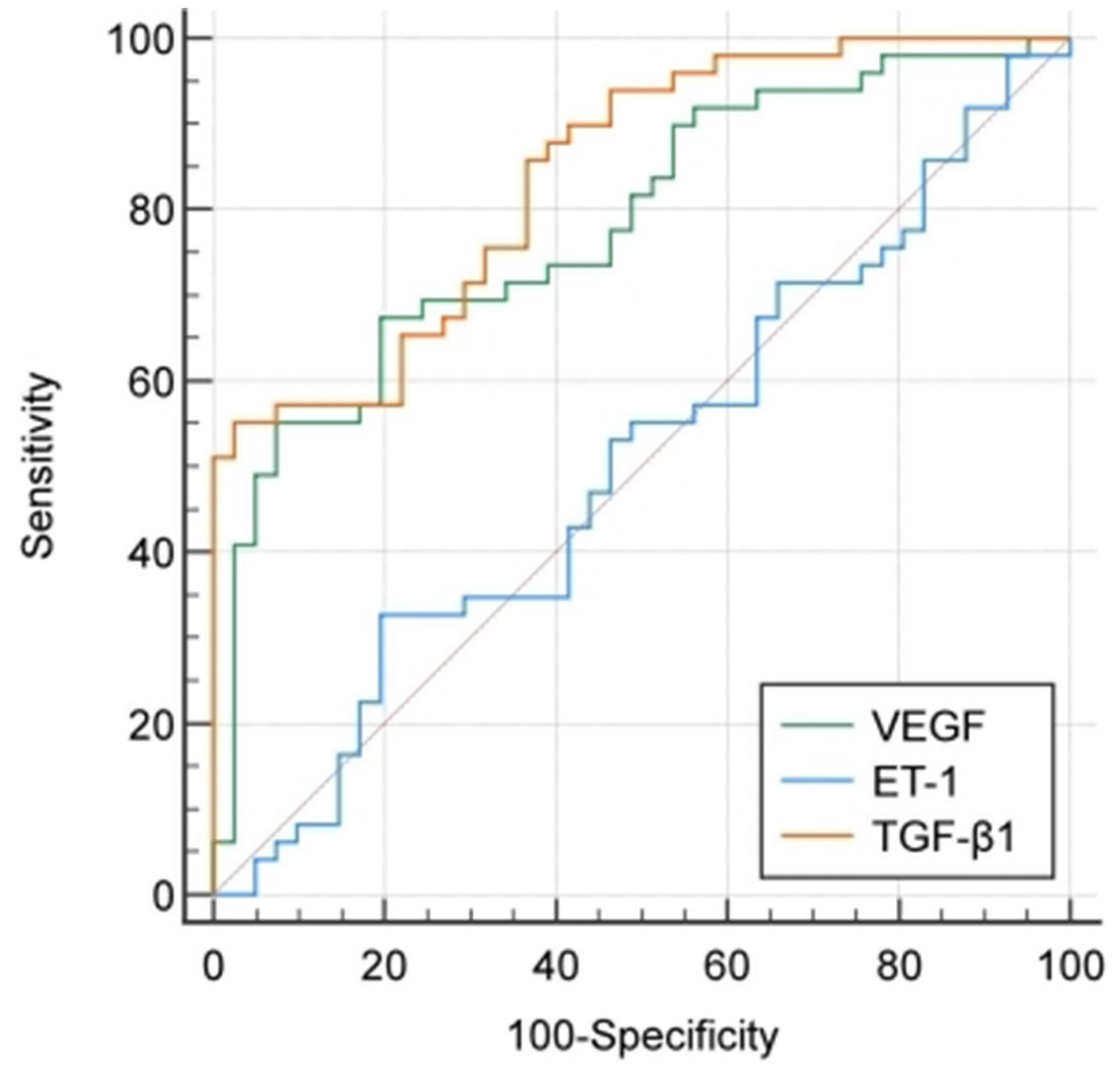
ROC curve analysis of VEGF, ET-1 and TGF-β1 levels in peritoneal dialysis effluent to predict peritoneal solute transport function.

The ROC curve analysis has demonstrated that the concentrations of VEGF and TGF-β1 present in the peritoneal dialysis effluent had a significant impact in predicting the peritoneal solute transport function (*p* < 0.05). Please refer to Table 4 and Figure 3.

**Table 4:** The predictive significance of the levels of VEGF, ET-1 and TGF-β1 in peritoneal dialysis effluent for the transport function of solutes in the peritoneum.

Indicators	Area under the curve	95 percent CI	*p*-value	Truncation value	Jordon index	Sensitivity (%)	Specificity (%)
VEGF	0.782	0.683–0.863	<0.001	>409.83 ng/mL	0.478	67.35	80.49
ET-1	0.507	0.399–0.614	0.914	≤262.34 pg/mL	0.131	32.65	80.49
TGF-β1	0.839	0.746–0.908	<0.001	>353.19 pg/mL	0.527	55.10	97.56

### 3.5 Analysis of prognosis

**Figure 4:**
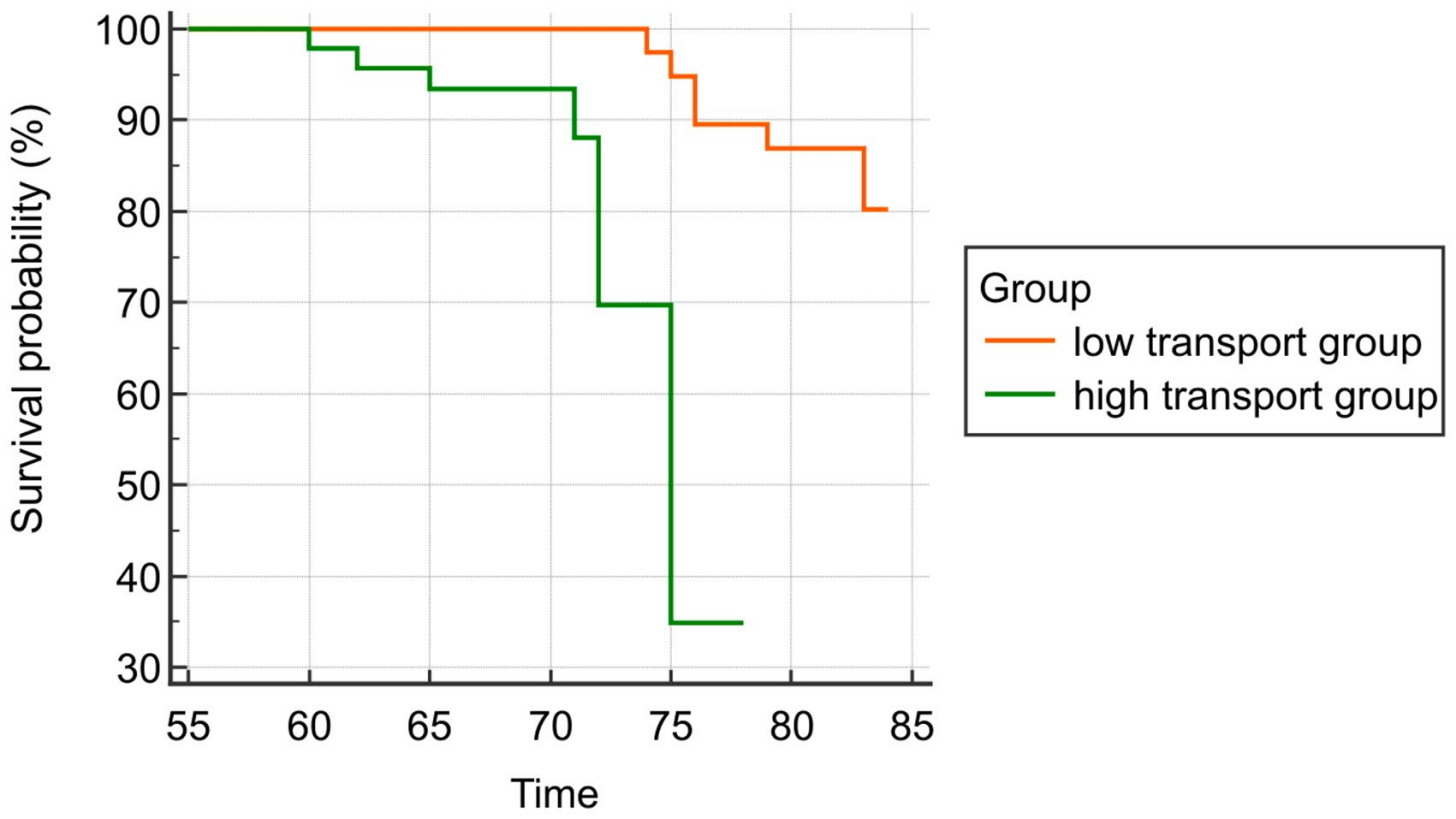
Prognostic analysis chart.

The median follow-up period for all patients was (34.63 ± 10.34) months, and 70 patients (15.56%) passed away from all causes. According to Kaplan–Meier survival analysis, the survival rate of the low transport group was observed to be higher than that of the high transport group (*p* < 0.05). Please refer to Figure 4.

### 3.6 Cox regression analysis

The Cox regression analysis indicated that the concentrations of VEGF and TGF-β1 present in peritoneal dialysis effluent were autonomous predictors of death in peritoneal dialysis patients (RR = 3.442, 3.550, *p* < 0.05). Please refer to Table 5.

**Table 5:** Multivariate cox regression analysis was used to analyze the prognostic factors.

Variant	Regression coefficient	Standard error	Wald *χ*^2^ value	*p*-value	RR value	95 per cent CI
VEGF	1.236	0.447	7.646	0.006	3.442	1.433 to 8.266
ET-1	0.617	0.403	2.344	0.127	1.853	0.841–4.083
TGF-β1	1.267	0.429	8.722	0.003	3.550	1.531–8.230

## 4 Discussion

With China’s rapidly aging population and shifting dietary patterns, the prevalence of chronic renal failure has escalated substantially, driving increased utilization of peritoneal dialysis (PD) as renal replacement therapy. This longitudinal exposure inevitably modifies the peritoneal membrane microenvironment, inducing both structural alterations such as angiogenesis and fibrosis, and functional impairments including elevated solute transport rates and diminished ultrafiltration capacity (13, 14). Chronic microinflammation in PD patients stems from persistent immune activation—where white blood cells, monocytes, and macrophages release cytokines—compounded by impaired renal clearance of uremic toxins and inflammatory mediators. These factors collectively trigger mesenchymal transition of peritoneal mesothelial cells, aberrant neovascularization, and progressive fibrosis, establishing a self-amplifying cycle of membrane dysfunction (15, 16).

Our multicenter retrospective analysis demonstrates that PD effluent biomarkers, particularly VEGF and TGF-β1, serve as critical mediators of peritoneal membrane dynamics. High-transport patients (D/Pcr > 0.65) exhibited significantly elevated VEGF and TGF-β1 levels compared to low-transport counterparts, with Pearson correlations confirming robust positive associations between biomarker concentrations and solute transport rates. Multivariate logistic regression further established VEGF (OR = 3.44; 95%CI 1.43–8.27) and TGF-β1 (OR = 3.55; 95%CI 1.53–8.23) as independent diagnostic determinants of transport status, corroborated by outstanding discriminatory power in ROC analysis (VEGF AUC = 0.782; TGF-β1 AUC = 0.839). Mechanistically, these cytokines operate synergistically: TGF-β1 initiates mesothelial-mesenchymal transition (MMT) through Smad2/3 signaling, converting mesothelial cells into profibrotic α-SMA + myofibroblasts (17, 18). These activated cells subsequently secrete VEGF, propagating angiogenesis via VEGFR-2 binding and directly enhancing vascular permeability—culminating in the peritoneal hyperpermeability that defines high-transport states (19, 20). TGF-β1 emerges as a critical pathological signaling hub in our analysis, demonstrating spatiotemporal regulation within the peritoneal membrane. Its submesothelial zone enrichment correlates with progressive collagen deposition, functioning through a dual-phase activation mechanism: initial induction is driven primarily by glucose degradation products in bioincompatible dialysate, while sustained amplification in advanced stages links to hypoxia from aberrant neovascularization. This temporal transition positions TGF-β1 beyond a mere injury-response mediator—it evolves into a self-perpetuating fibrosis amplifier through autocrine myofibroblast activation and paracrine crosstalk with vascular mediators. Critically, the spatial coexistence of TGF-β1 hotspots with fibronectin-rich domains and its bidirectional reinforcement of vascular endothelial growth signaling create therapeutic-resistant microenvironments. These findings elucidate the limitations of prior glucose-sparing monotherapies and establish a mechanistic foundation for combinatorial strategies concurrently targeting TGF-β1 signaling and vascular stabilization (3, 21, 22). This pathophysiological cascade explains the dramatic structural reorganization observed in long-term PD patients, including collagen deposition, expanded vascular networks, and mesothelial injury. Notably, ET-1 demonstrated neither intergroup differences nor transport correlations, consistent with its molecular size limiting peritoneal transit (23). This observation directly refutes hypothesized ET-1/lymphangiogenesis links while underscoring the primacy of VEGF/TGF-β1 pathways in solute transport dysregulation (24–26).

Clinically, our findings reveal grave prognostic implications. Kaplan–Meier analysis demonstrated a 35% absolute reduction in 5-year survival among high-transport patients, with multivariate Cox regression confirming VEGF and TGF-β1 as independent mortality predictors. This survival deficit primarily stems from ultrafiltration failure—manifesting as volume overload and profound protein loss (13 g/L dialysate albumin)—which affected over 60% of high-transport patients during the 34.6 ± 10.3 month observation period. These biomarkers’ predictive value extends beyond mortality, offering sensitive operational thresholds: VEGF > 409.83 ng/mL achieved 67.35% sensitivity and 80.49% specificity, while TGF-β1 > 353.19 pg/mL demonstrated 55.10% sensitivity and exceptional 97.56% specificity for identifying high-transport membranes. These outcomes originate in interrelated pathological cascades: bioincompatible dialysates and retained uremic toxins perpetuate macrophage activation, driving TNF-α and IL-6 release that amplifies VEGF/TGF-β1 expression. The resultant neovascularization expands capillary surface area, accelerating solute clearance while paradoxically impairing ultrafiltration through heightened endothelial porosity. This self-perpetuating cycle—where hyperpermeability promotes mesothelial transition-mediated fibrosis, and fibrotic distortion subsequently amplifies permeability—explains the accelerated technique failure observed in high-transport phenotypes (27–29).

Our findings mandate clinical paradigm shifts. First, VEGF/TGF-β1 assay integration into routine PD monitoring enables preemptive high-transport identification before overt ultrafiltration failure. Second, our data validate targeted interventions: TGF-β1 inhibitors (e.g., pirfenidone) demonstrated antifibrotic efficacy in preclinical models (30), while VEGF antagonists like bevacizumab reduced angiogenesis in clinical PD cohorts (31). Third, protocol adjustments during follow-up—shifting to icodextrin solutions, intensifying volume management, or nutritional modifications—may mitigate membrane deterioration. Crucially, these biomarkers facilitate precise risk-stratification: patients exceeding identified thresholds warrant aggressive monitoring and early intervention.

### 4.1 Limitations and future directions

Despite the significant findings, this study has some limitations. First, as a retrospective study, it relies on existing medical records, which may contain incomplete or inaccurate data. While causes of ESRD and genetic variability (%) represent valuable information, these parameters were not consistently captured in the original retrospective medical records analyzed for this study. Secondly, although it is a multicenter study, the sample size is relatively limited, which may affect the generalizability of the results. Third, limited retrospective capture of granular biochemical profiles-particularly inflammation mediators (TNF-α, IL-6) and cardiac stress biomarkers (NT-proBNP)-precluded comprehensive pathway dissection. This reflects pragmatic constraints in harmonizing historical assays across diverse hospital laboratories where such measurements followed institution-specific clinical indications rather than standardized research protocols. Additionally, this study primarily focused on VEGF and TGF-β1 levels, without comprehensively exploring other factors that might influence peritoneal transport function.

Future research should expand the sample size and include prospective studies to validate these findings. Moreover, other biomarkers and mechanisms should be explored to gain a deeper understanding of the regulation of peritoneal transport function. To address biochemical depth limitations, our ongoing prospective cohort implements: (a) Centralized core laboratory processing with scheduled biobanking at Months 0/6/12; (b) Systematic capture of arterial stiffness indices (cfPWV) and iterative hemofiltration effluent kinetics linked to ASCVD risk panels; and (c) Embedded metabolomic sub-study profiling lipidomics and uremic toxins via LC–MS. This layered approach will transcend current constraints by establishing temporal biomarker trajectories while defining specific thresholds for adiponectin, oxidized LDL, and fibroblast growth factor-23 with therapeutic relevance. Developing new therapeutic strategies to modulate VEGF and TGF-β1 levels and prevent or reverse peritoneal fibrosis will be a crucial direction for future research. Through these efforts, we aim to further improve the prognosis and quality of life for peritoneal dialysis patients.

## 5 Summary

This multicenter retrospective study, conducted by four institutions, reveals the significant impact of peritoneal transport function on the prognosis of long-term peritoneal dialysis patients. The results indicate that patients with high peritoneal transport function have a lower survival rate compared to those with low transport function. Moreover, levels of VEGF and TGF-β1 in peritoneal dialysis effluent were identified as independent factors influencing peritoneal solute transport function. Kaplan–Meier survival analysis and COX regression analysis further confirmed that VEGF and TGF-β1 levels are crucial for predicting the prognosis of peritoneal dialysis patients. These findings not only help in identifying high-risk patients but also provide important biomarkers for clinical intervention.

## Data Availability

The raw data supporting the conclusions of this article will be made available by the authors, without undue reservation.
